# Research on the metabolic regulation mechanism of Yangyin Qingfei decoction plus in severe pneumonia caused by *Mycoplasma pneumoniae* in mice

**DOI:** 10.3389/fphar.2024.1376812

**Published:** 2024-04-17

**Authors:** Tianyu Zhang, Xiyu Zhao, Xining Zhang, Xiangyu Liang, Zhenglong Guan, Guanghan Wang, Guanghua Liu, Zhenqi Wu

**Affiliations:** ^1^ The First Clinical College of Liaoning University of Traditional Chinese Medicine, Shenyang, China; ^2^ The Second Affiliated Hospital of Liaoning University of Traditional Chinese Medicine, Shenyang, China; ^3^ College of Traditional Chinese Medicine, Liaoning University of Traditional Chinese Medicine, Shenyang, China

**Keywords:** Yangyin Qingfei decoction plus, *Mycoplasma pneumoniae*, severe illness, metabolomics, PI3K/Akt/NF-κB

## Abstract

**Introduction:** With amazing clinical efficacy, Yangyin Qingfei Decoction Plus (YQDP), a well-known and age-old Chinese compound made of ten Chinese botanical drugs, is utilized in clinical settings to treat a range of respiratory conditions. This study examines the impact of Yangyin Qingfei Decoction (YQDP) on lung tissue metabolic products in severe Mycoplasma pneumoniae pneumonia (SMPP) model mice and examines the mechanism of YQDP in treating MP infection using UPLC-MS/MS technology.

**Methods:** YQDP’s chemical composition was ascertained by the use of Agilent 1260 Ⅱ high-performance liquid chromatography. By using a nasal drip of 10^10^ CCU/mL MP bacterial solution, an SMPP mouse model was created. The lung index, pathology and ultrastructural observation of lung tissue were utilized to assess the therapeutic effect of YQDP in SMPP mice. Lung tissue metabolites were found in the normal group, model group, and YQDP group using UPLC-MS/MS technology. Using an enzyme-linked immunosorbent test (ELISA), the amount of serum inflammatory factors, such as interleukin-6 (IL-6) and tumor necrosis factor α (TNF-α), was found. Additionally, the protein expression of PI3K, P-PI3K, AKT, P-AKT, NF-κB, and P-NF-κB was found using Western blot.

**Results:** The contents of chlorogenic acid, paeoniflorin, forsythrin A, forsythrin, and paeonol in YQDP were 3.480 ± 0.051, 3.255 ± 0.040, 3.612 ± 0.017, 1.757 ± 0.031, and 1.080 ± 0.007 mg/g respectively. YQDP can considerably lower the SMPP mice’s lung index (*p* < 0.05). In the lung tissue of YQDP groups, there has been a decrease (*p* < 0.05) in the infiltration of inflammatory cells at varying concentrations in the alveoli compared with the model group. A total of 47 distinct metabolites, including choline phosphate, glutamyl lysine, L-tyrosine, 6-thioinosine, Glu Trp, 5-hydroxydecanoate, etc., were linked to the regulation of YQDP, according to metabolomics study. By controlling the metabolism of porphyrins, pyrimidines, cholines, fatty acids, sphingolipids, glycerophospholipids, ferroptosis, steroid hormone biosynthesis, and unsaturated fatty acid biosynthesis, enrichment analysis suggested that YQDP may be used to treat SMPP. YQDP can lower the amount of TNF-α and IL-6 in model group mice as well as downregulate P-PI3K, P-AKT, and P-NF-κB expression (*p* < 0.05).

**Conclusion:** A specific intervention effect of YQDP is observed in SMPP model mice. Through the PI3K/Akt/NF-κB signaling pathways, YQDP may have therapeutic benefits by regulating the body’s metabolism of α-Linoleic acid, sphingolipids, glycerophospholipids, arachidonic acid, and the production of unsaturated fatty acids.

## 1 Introduction

One of the most frequent infections in children with community-acquired pneumonia (CAP) is *Mycoplasma* pneumoniae (MP), which has a widespread pathogenicity and frequently exhibits periodic and regional epidemiology. Ten to forty percent of pediatric pneumonia cases are caused by *Mycoplasma* pneumoniae pneumonia (MPP), which is the result of MP infection (Kutty et al., 2019). The MP infection is typically regarded as a self-limiting illness. It is marked by subtle respiratory symptoms, the most notable of which is paroxysmal dry cough, which is frequently followed by the expectoration of little amounts of mucus or mucopurulent sputum. Although MPP is thought to be self-limiting, 0.5%–to 2.0% of instances are known to proceed to severe MPP (SMPP). This condition is linked to life-threatening consequences such respiratory failure and acute respiratory distress syndrome, which can be fatal (Izumikawa, 2016; Lee et al., 2021). Although the exact pathogenic mechanism of MP infection is unknown, some studies think that adhesion and cytotoxic effects cause direct damage to the respiratory epithelium during MP pathogenesis (Atkinson et al., 2008). When MP infection damages host mucosal epithelial cells, it can generate toxic compounds that cause the epithelial cells to die and apoptose, which can result in pneumonia and other extrapulmonary injuries (Sun et al., 2008; He et al., 2016). Since MP lacks a cell wall structure, it is resistant to antibiotics that stop bacteria from producing cell walls, but it is susceptible to quinolones, tetracyclines, and macrolides. For this reason, macrolides are the recommended treatment for MPP in children. Unfortunately, resistance to macrolides has grown in many regions since the discovery of macrolide-resistant strains of MP in China in 2003. Additionally, the side effects of macrolide antibiotics, such as gastrointestinal distress and vascular irritation, have made the hunt for more effective treatment options necessary (Xin et al., 2005; Zhao et al., 2019; Dou et al., 2020). There are benefits to using traditional Chinese medicine (TCM) in the treatment of SMPP, and research on the targeted mechanism of TCM intervention in SMPP is currently a popular topic.

The “Chonglou Yuyao” book, authored by renowned Qing Dynasty physician Zheng Meijian, is where Yangyin Qingfei Decoction Plus (YQDP) originated. Ten traditional Chinese medicines are included in it: Rehmanniae Radix, Scrophulariae Radix, Ophiopogonis Radix, Paeoniae Radix Alba, Moutan Cortex, Fritillariae Thunbergii Bulbus, Menthae Haplocalycis Herba, Glycyrrhizae Radix Et Rhizoma, Forsythiae Fructus, and Lonicerae Japonicae Flos. In clinical settings, YQDP is frequently used to treat bronchopneumonia, post-infection cough, and chronic obstructive pulmonary disease, among other conditions (Liu et al., 2017; Min et al., 2021). Contemporary pharmacological studies have demonstrated that YQDP functions by increasing tissue healing, controlling immunological function, and suppressing inflammatory reactions (Tang et al., 2020). Forsythoside, a representative of Forsythiae Fructus, has shown antiviral, anti-inflammatory, and antioxidant properties in contemporary studies (Ma et al., 2020). Phenolic acid compounds in Lonicerae Japonicae Flos have pharmacological activities such as antiviral, antibacterial, anti-inflammatory, and antioxidant effects. Compounds containing phenolic acid in Lonicerae Japonicae Flos include antioxidant, antiviral, and antibacterial properties (Xiong et al., 2022). As an indication component of Lonicerae Japonicae Flos, chlorogenic acid possesses a variety of anti-inflammatory properties (Gao et al., 2019) and can eliminate reactive oxygen species within cells (Shin et al., 2017; Liang and Kitts, 2018). The primary ingredients of Forsythiae Fructus, forsythiaside A and forsythin, have potent antiviral and anti-inflammatory properties (Qu et al., 2016; Runfeng et al., 2020). The functional elements of Moutan Cortex and Paeoniae Radix Alba are pheonol and pheoniflorin, respectively. The analgesic and anti-inflammatory properties of monoterpenes and their glycosides, embodied by Paeoniflorin, are acknowledged as the pharmacologically active components of Paeoniae Radix Alba (Wu et al., 2018; Zhang et al., 2021). Paeonol has the ability to protect against oxidative stress, prevent the production of reactive oxygen species, and boost the activity of the enzymes superoxide dismutase and hydrogen peroxide (Zhang et al., 2020). In earlier research, we established and evaluated a severe pneumonia model in high-load MP-infected mice (Wu et al., 2023a) and showed that YQDP regulates the TLR2/MyD88/NF-κB signaling pathway, Aquaporin5 (AQP5), and Mucin 5ac (MUC5ac) to regulate SMPP (Wu et al., 2023b; Qi et al., 2023).

An HPLC method is used in this study to simultaneously determine the level of five useful compounds in YQDP for quality control. The therapeutic benefits and metabolic pathways of YQDP on SMPP were then examined.

## 2 Materials and methods

### 2.1 Reagents and chemicals

Merck Company (USA) provided the acetonitrile (CAS: 75-05-8), phosphoric acid (CAS: 7664-38-2), and methanol (CAS: 67-56-1). The China Academy of Food and Drug Administration provided the following: Chlorogenic Acid (CGA, CAS: 327-97-9), Paeoniflorin (PF, CAS: 23180-57-–6), Forsythoside A (FTA, CAS: 79916-77-1), Forsythin (FT, CAS: 487-41-2), and Paeonol (PAE, CAS: 552-41-0). Proteintech was the source of the following antibodies: Anti-GAPDH (60004-1-Ig), Anti-PI3K (60225-1-Ig), Anti-AKT (10176-2-AP), Anti-Phospho-AKT (66444-1-Ig), and Anti-NFκB p65 (10745-1-AP). Bioss provided the anti-phospho-PI3K (bs-3332R) and anti-phospho-NFκB p65 (bs-3543R) antibodies.

*Rehmannia glutinosa* (Gaertn.) DC. [Orobanchaceae] (Rehmanniae Radix, 2202163), *Scrophularia ningpoensis* Hemsl. [Scrophulariaceae] (Scrophulariae Radix, 2203081), *Ophiopogon japonicus* (Thunb.) Ker Gawl. [Asparagaceae] (Ophiopogonis Radix, 2203052), *Paeonia lactiflora* Pall. [Paeoniaceae] (Paeoniae Radix Alba, 2203152), *Paeonia × suffruticosa* Andrews [Paeoniaceae] (Moutan Cortex, 2,203,121), *Fritillaria thunbergii* Miq. [Liliaceae] (Fritillariae Thunbergii Bulbus, 2203151), *Mentha canadensis* L. [Lamiaceae] (Menthae Haplocalycis Herba, 2203241), *Glycyrrhiza uralensis* Fisch. ex DC. [Fabaceae] (Glycyrrhizae Radix Et Rhizoma, 2203152), *Forsythia suspensa* (Thunb.) Vahl [Oleaceae] (Forsythiae Fructus, 2204102), and *Lonicera japonica* Thunb. [Caprifoliaceae] (Lonicerae Japonicae Flos, 2203081) were purchased from Anhui Puren Traditional Chinese Medicine Drinking Tablets Co Ltd (Anhui, China) and authenticated by Professor Leng Yujie from the Chinese Herbal Medicine Bureau of the Affiliated Hospital of Liaoning University of Traditional Chinese Medicine (Shenyang, China). Rehmanniae Radix sample (RGL20220617), Scrophulariae Radix sample (SNH20220618), Ophiopogonis Radix sample (OJK20220619), Paeoniae Radix Alba sample (PLP20220617), Moutan Cortex sample (PSA20220617), Fritillariae Thunbergii Bulbus sample (FTM20220617), Menthae Haplocalycis Herba sample (MHB20220617), Glycyrrhizae Radix Et Rhizoma sample (GUF20220617), Forsythiae Fructus sample (FSV20220617) and Lonicerae Japonicae Flos sample (LJT20220617) were kept in the Laboratory of Pharmacognosy, Liaoning Academy of Traditional Chinese Medicine.

### 2.2 Experimental animals and strains

36 SPF BALB/c mice, 4–6 weeks, weighing 20 ± 2g, were provided by Liaoning Changsheng Biotechnology Co., Ltd. [SCXK (Liao) 2020–0001]. The mice were kept in separate cages at the Experimental Animal Center of Liaoning University of Traditional Chinese Medicine [SYXK (Liao) 2019-004]. The housing environment was kept at (22 ± 2)°C, with a 12h/12 h light/dark cycle, and a humidity of (50 ± 2)%. The MP standard strain FH was stored in the virus room of the Affiliated Hospital of Liaoning University of Traditional Chinese Medicine. The Animal Ethics Committee of Liaoning University of Traditional Chinese Medicine gave its approval to this study (Approval No.: 21000042021131).

### 2.3 Determination of five active ingredients in YQDP

#### 2.3.1 Preparation of standard solutions

Precisely weighing CGA, PF, FTA, FT, and PAE, then dissolving them with methanol to create a solution with CGA at 0.214 mg/mL, PF at 0.217 mg/mL, FTA at 0.214 mg/mL, FT at 0.118 mg/mL, and PAE at 0.068 mg/mL.

#### 2.3.2 Preparation of YQDP sample solutions

The procedure described in the literature was used to make YQDP (Wu et al., 2019). Rehmanniae Radix (15 g), Scrophulariae Radix (11 g), Ophiopogonis Radix (9 g), Paeoniae Radix Alba (6 g), Moutan Cortex (6 g), Fritillariae Thunbergii Bulbus (6 g), Menthae Haplocalycis Herba (4 g), Glycyrrhizae Radix Et Rhizoma (4 g), Forsythiae Fructus (6 g) and Lonicerae Japonicae Flos (6 g) were then placed in a 10-fold 70% ethanol (v/v). The extract was then extracted using cold reflux for 70 min, filtered, concentrated to create a paste, and dried at a constant temperature to produce YQDP dry powder. Ultimately, 25 mL of methanol and 0.5 g of the dry powder were combined, sonicated, and then filtered through a 0.45 µm filter membrane for additional usage.

#### 2.3.3 HPLC analysis

A SHIMADZU-GL C18 (250 mm × 4.6 mm × 5 μm) column was used in an Agilent 1260 high-performance liquid chromatograph to determine the content. The flow rate was 0.8 mL/min and the column temperature was 30°C. The following gradient elution conditions applied to the mobile phase, which was 0.1% phosphoric acid-water (A) and acetonitrile (B): 0∼20 min, 10%∼20% (B); 20∼25 min, 12%∼15% (B); 25∼30 min, 15%–18% (B); 30–50 min, 18%–36% (B); 50∼70 min, 36%∼50% (B); 70∼75 min, 50%∼50% (B); 75∼80 min, 50%∼60% (B); 80∼85 min, 60%∼70% (B). The following were the detecting wavelength and program: 0∼25 min, 330 nm; 25∼37 min, 230 nm; 37∼45 min, 280 nm. 5 μL was the injection volume of the sample.

### 2.4 Preparation of YQDP prescription for intervention in SMPP model mice

Condensation and refluxing with 70% ethanol for 70 min was used to extract Rehmanniae Radix (150 g), Scrophulariae Radix (110 g), Ophiopogonis Radix (90 g), Paeoniae Radix Alba (60 g), Moutan Cortex (60 g), Fritillariae Thunbergii Bulbus (60 g), Menthae Haplocalycis Herba (40 g), Glycyrrhizae Radix Et Rhizoma (40 g), Forsythiae Fructus (60 g) and Lonicerae Japonicae Flos (60 g). 176.23 g of the extract were obtained by drying the extracts at a steady temperature after they had been concentrated into a paste. The drug extraction rate was 24.14%.

### 2.5 MP cultivation

After thawing under running water, the MP strain was introduced to PPLO liquid medium and cultured at 37°C in an incubator set to a constant temperature with 5% CO_2_. The third generation was removed and placed aside when the liquid medium turned yellow instead of red. The Color Change Unite (CCU) was defined as the highest dilution concentration at which the medium turned yellow. The MP concentration was ascertained using the CCU/mL method, and the gradient dilution method was used to prepare the bacterial solution with a concentration of 1×10^10^ CCU/mL.

### 2.6 Animal grouping and dosing

Following a week of consistent adaptive feeding, 36 BALB/c mice were split into six groups at random (*n* = 6). Referring to the modeling method of previous studies (Wu et al., 2023a), mice were anesthetized with isoflurane. Mice in the normal group were given nasal drip of 100 μL of physiological saline, while mice in the other groups were given nasal drip of 100 μL of MP bacterial solution at a concentration of 1×10^10^ CCU/mL. All groups received nasal drip for three consecutive days.

According to the “Animal Dose Conversion Table” (Wei, 2010), the positive group was administered azithromycin dry suspension at a total dose of 0.370 g/kg. The YQDP group received three dosages of formula solution (1.552, 3.104, 6.208 g/kg, crude drug), The normal group and model group were given an equal amount of physiological saline. Lung tissue was utilized for pathological and metabolomic research, and serum from each group of mice was obtained for ELISA detection following continuous dosing for 7 days.

### 2.7 Mouse lung index

To determine the lung index, lung tissues were blotted dry on filter paper, washed with 0.9% saline, and then weighed.

$$\text{Lung index}=\left(\text{lung weight }/\text{ body weight}\right)\times 100\%$$

### 2.8 Histopathological observation

After being fixed with 4% paraformaldehyde, the lung tissue was sectioned into thin 5 μm slices using a sectioning machine and embedded into wax blocks using a paraffin embedding machine. Under a light microscope, the pathological alterations in the lung tissue following HE staining were seen.

### 2.9 Observation of lung tissue ultrastructure

Two to three pieces of lung tissue (1 mm^3^) were removed from each group, preserved using 2.5% glutaraldehyde and 1% osmium acid, gradually dried with acetone, and then sliced using an epoxy resin infiltration and embedding process. The samples were then stained with lead citrate and uranyl acetate so that an H-7650 transmission electron microscope could be used to observe the ultrastructural alterations in lung tissue.

### 2.10 Metabolomics analysis

0.1 g of lung tissue samples were weighed and then finely powdered using liquid nitrogen. Each group of samples received four times the volume of the extraction buffer MeOH/ACN (1:1, v/v), which was added, properly vortexed, and then incubated at −20°C for 1 h before being centrifuged at 18,000 g for 15 min at 4 °C. After transferring the liquid metabolite extraction supernatant to a centrifuge tube, separate an aliquot of 10 μL dilution solution from each sample to combine with quality control samples in preparation for metabolomics analysis. Utilizing the Waters ACQUITY UPLC ultra-performance liquid chromatography system in conjunction with the Waters ACQUITY UPLC BEH C18 Column (1.7 µm, 2.1 mm × 100 mm) for separation, 10 µL of injection volume, 0.40 mL/min of elution, and 40°C column temperature, the analysis was carried out.

The mobile phase A was an aqueous solution containing 0.1% formic acid, and the mobile phase B was an acetonitrile-water solution containing 0.1% formic acid. The liquid phase gradient was set as follows: 0–11 min, 2%–98% B; 11–12 min, 98% B; 12–12.1 min, 98%–2% B; 12.1–15 min, 2% B. The metabolites were separated by an ultra-high-performance liquid-phase system and injected into an ESI ion source for ionization and then analyzed by timsTOF Pro (Bruker) mass spectrometry. The ion source voltage was set at 4.5 kV, and both precursor ions and its secondary fragments were detected and analyzed using high-resolution TOF. The mass spectrometry scan range was set at 50–1,300 m/z, and the data acquisition mode used Parallel Accumulation Serial Fragmentation (PASEF) mode.

### 2.11 Enzyme-linked immunosorbent assay (ELISA) for the detection of IL-6 and TNF-α expression in serum

The serum was divided into sterile EP tubes, centrifuged for 20 min at 3,000 r/min, and refrigerated at −80°C. Following the kit’s instructions, add the sample, enzyme-labeled antibody, chromogenic reagent, and stop solution once the reagents have warmed up to room temperature. Then, use a 450 nm wavelength to examine the contents.

### 2.12 Protein expression of PI3K, P-PI3K, AKT, P-AKT, NF-κB, P-NF-κB in lung tissues by Western blot

After extracting the total protein from the lung tissue, the concentration of the protein was measured quantitatively and adjusted to a uniform concentration. 10% SDS-PAGE gel electrophoresis was used for protein separation, and the wet transfer technique was used to transfer the proteins to a PVDF membrane. The primary antibodies, PI3K (1:2000), P-PI3K (1:2000), AKT (1:10,000), P-AKT (1:5000), NF-κB (1:5000), P-NF-κB (1:5000), and the internal reference antibody GAPDH (1:10,000), were incubated after blocking with 5% non-fat milk powder. At 4°C, the incubation was carried out all night. The membrane was washed three times with TBST and then incubated with HRP-conjugated goat anti-rabbit secondary antibody (1:10,000) at room temperature for 1 h. Following membrane cleaning, the PVDF membrane was put in a chemiluminescence imaging system for detection after the proper volume of ECL luminous solution was introduced. The internal control protein’s grayscale value served as a mathematical reference while using ImageJ software for analysis.

### 2.13 Statistical analysis

The statistical software GraphPad Prism six was used for both graph processing and statistical analysis. When it came to quantitative data that satisfied the requirements for normality and homogeneity of variance, the one-way ANOVA test was employed, and the data were reported as Mean ± SD. *p < 0.05 was considered statistically significant.

## 3 Results

### 3.1 HPLC determination of five chemical components in YQDP

**FIGURE 1:**
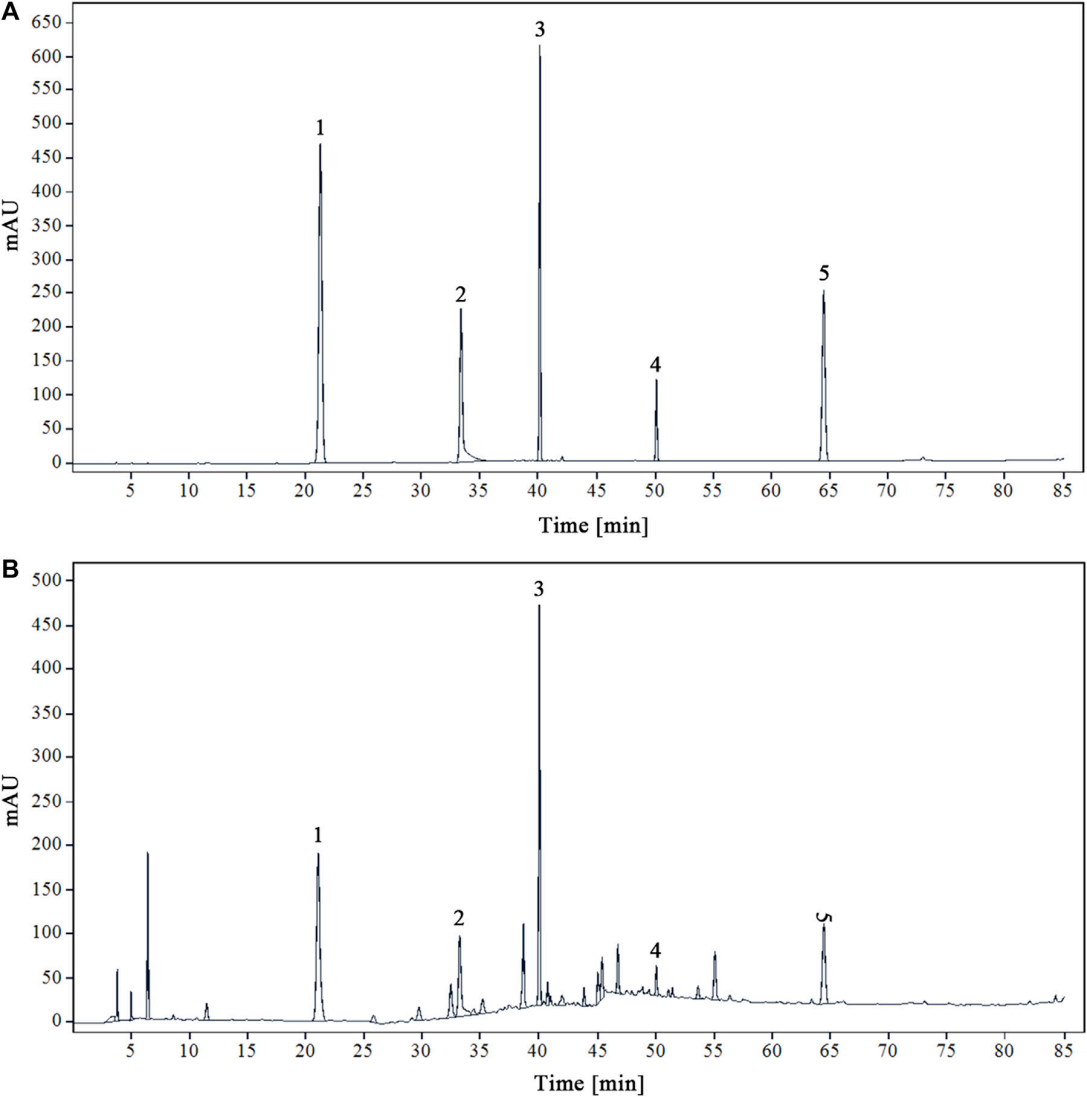
HPLC chromatograms of mixed reference solution **(A)** and modified sample solution of YQDP decoction **(B)** 1. CGA. 2. PF. 3. FTA. 4. FT. 5. PAE.

Using acetonitrile (B) and 0.1% phosphoric acid water (A) as mobile phases will yield the best peak shape and resolution. Figure 1 displays the chromatograms of the YQDP sample solution and the mixed reference solution under these circumstances.

### 3.2 Validation

Tests on linearity, accuracy, stability, reproducibility, and sample recovery identified five chemical components in the YQDP recipe. Supplementary Table S1 displays all of the component standard curves. According to the data, there is a good linear association (r ≥ 0.9996) between the compounds that were tested and their corresponding concentration ranges. CGA, PF, FTA, FT, and PAE have respective RSD between 0.31% and 0.88%. Within 48 h, all components exhibited good stability, with an RSD ranging from 0.56% to 1.25%. consistency, ranging from 0.47% to 1.79%. All components had recovery rates between 98.63% and 100.04%, with an RSD between 0.27% and 1.84%.

### 3.3 Content of five chemical components in YQDP

YQDP recipe’s five chemical components’ contents were ascertained by the application of the established analytical method. Table 1 displays the results.

**TABLE 1:** Content determination results of five chemical components in YQDP (mg/g, Mean ± SD, *n* = 6).

Component	CGA	PF	FTA	FT	PAE
Content	3.480 ± 0.051	3.255 ± 0.040	3.612 ± 0.017	1.757 ± 0.031	1.080 ± 0.007

### 3.4 Changes in lung index of mice in each group

**FIGURE 2:**
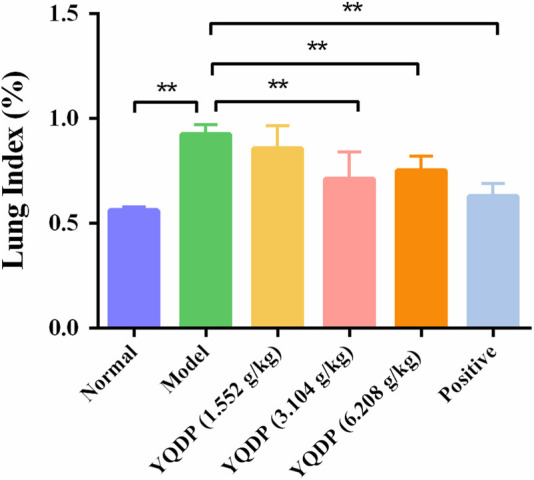
Lung index. **p < 0.01.

The lung index of the model group mice was higher than that of the normal group on the seventh day following MP infection (*p* < 0.01). At every dosage, YQDP groups and the positive group shown a decline in comparison to the model group. Both the YQDP (3.104 g/kg) and YQDP (6.208 g/kg) groups saw a considerable decline. These two YQDP groupings are not significantly different from one another. The outcomes are displayed in Figure 2.

### 3.5 Pathological observation and scoring of lung tissue

**FIGURE 3:**
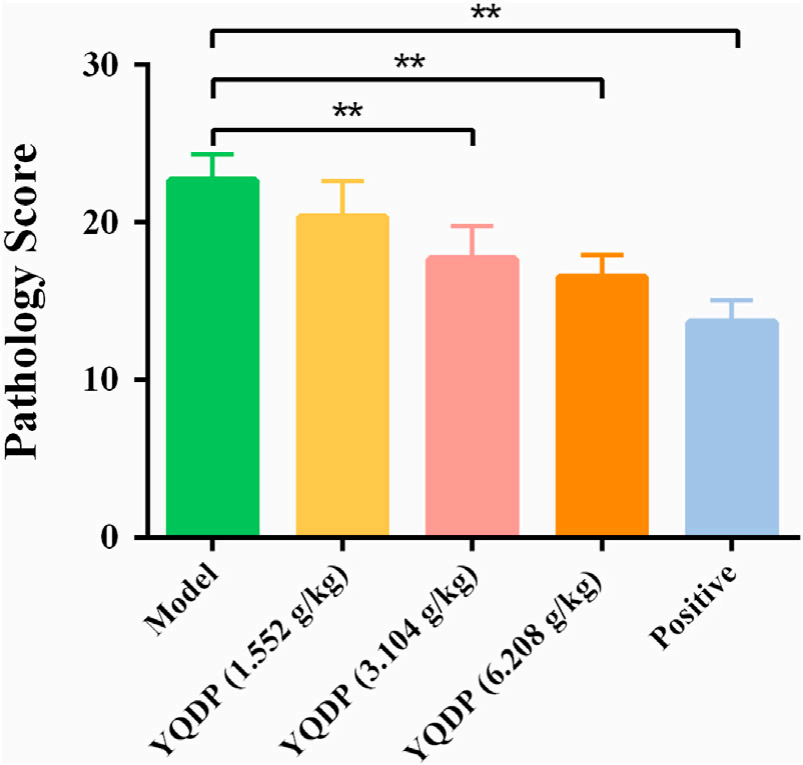
Pathological score. **p < 0.01.

**FIGURE 4:**
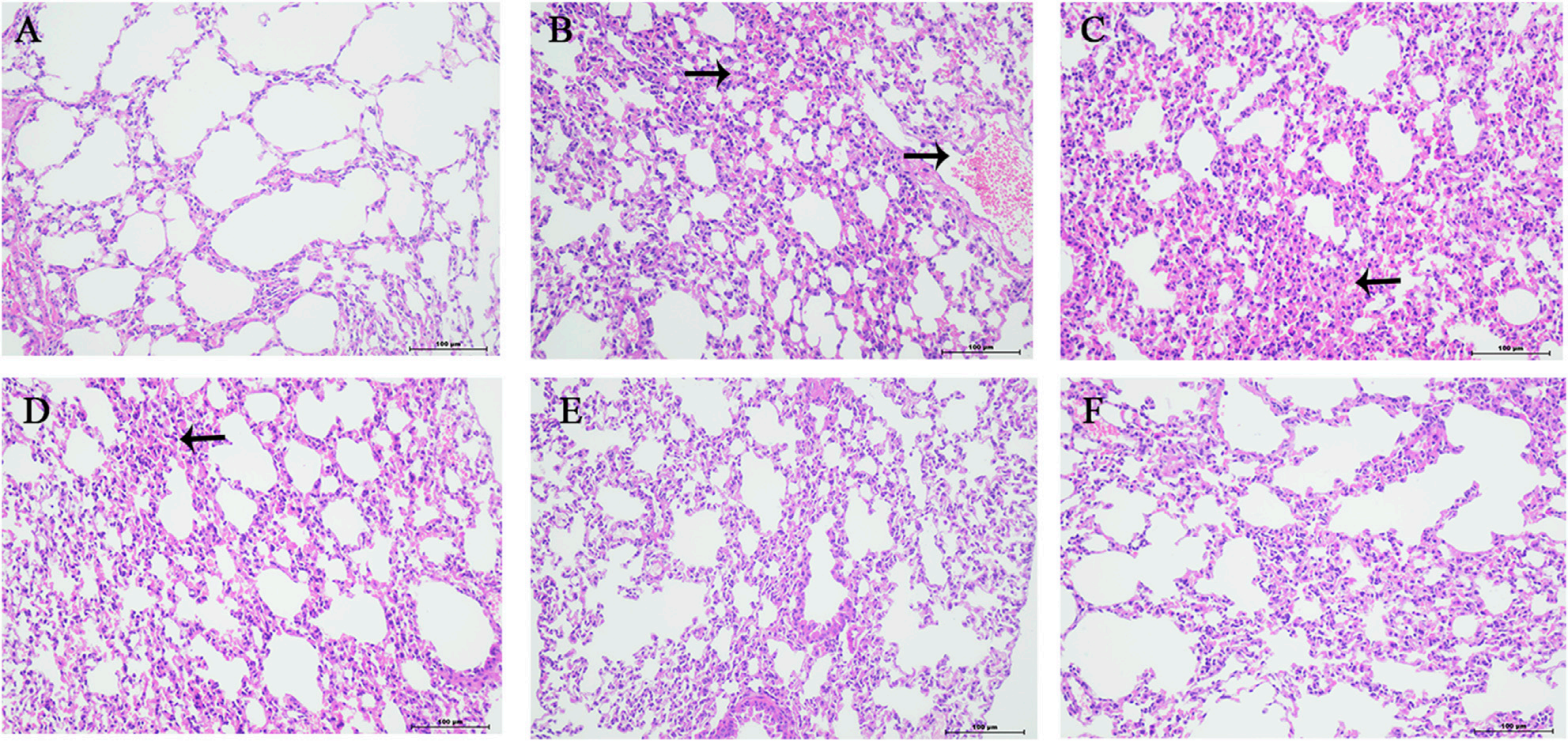
Pathology of lung tissue (HE, 200×). **(A)** Normal group. **(B)** Model group. **(C)** YQDP (1.552 g/kg). **(D)** YQDP (3.104 g/kg) group. **(E)** YQDP (6.208 g/kg) group. **(F)** Positive group.

The normal group mice had intact lung tissue morphology and structure, with no edema or congestion in the tracheal wall, and no infiltration of inflammatory cells in the surrounding blood vessels. Most of the alveolar structures in model group mice were severely damaged, with fewer intact alveoli, significantly widened alveolar septa, and a large number of inflammatory cells exuding from the alveolar lumen. Inflammatory cell exudation and widened alveolar septa were observed in lung tissue of YQDP group mice at various concentrations. Lymphocyte infiltration was observed around the bronchioles and blood vessels. The positive group mice showed inflammatory cell infiltration around the trachea, bronchi, and small blood vessels, with mild pulmonary tissue congestion. Figure 3 The results of the pathological score indicate that the lung tissue in the model group of mice had higher pathological scores than each treatment group (*p* < 0.01). The positive group performed better than the YQDP groups at different concentrations (*p* < 0.01). The outcomes are displayed in Figure 4.

### 3.6 Ultrastructural observation of lung tissue

**FIGURE 5:**
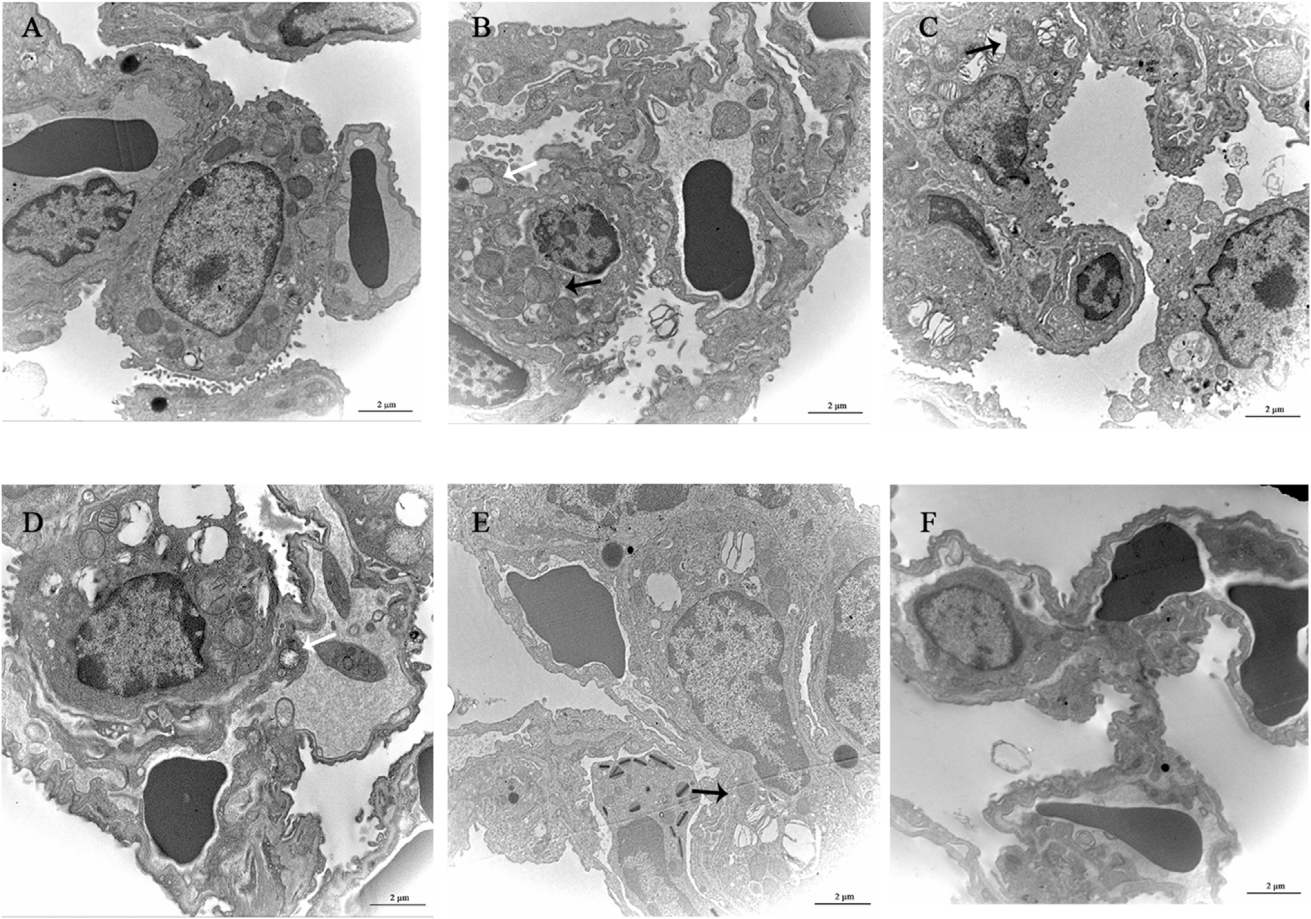
Observation of lung tissue ultrastructure (TEM, 120,00×). **(A)** Normal group. **(B)** Model group. **(C)** YQDP (1.552 g/kg). **(D)** YQDP (3.104 g/kg) group. **(E)** YQDP (6.208 g/kg) group. **(F)** Positive group. Lamina (White →), mitochondria (black →).

The alveolar wall of the normal group mice is intact, the cytoplasm is uniform, the nuclear membrane is intact, and a small amount of microvilli can be seen on the free surface. The microvilli on the free surface of type II alveolar cells in the model group mice thickened and disordered, with mitochondrial swelling and thickening of the thin layer of connective tissue and basement membrane between alveoli. YQDP groups of mice at different concentrations showed more microvilli on the surface of type II alveolar cells and clear nuclear membranes. There are organelles in the cytoplasm, such as mitochondria with clear cristae and numerous lamellar bodies, and there are cell junctions between adjacent cells. The microvilli in the positive group were slightly thickened, with a small amount of shedding and irregular arrangement. Some mitochondria were swollen, and the connective tissue and basement membrane were thickened. Results are shown in Figure 5.

### 3.7 Metabolomics analysis

**FIGURE 6:**
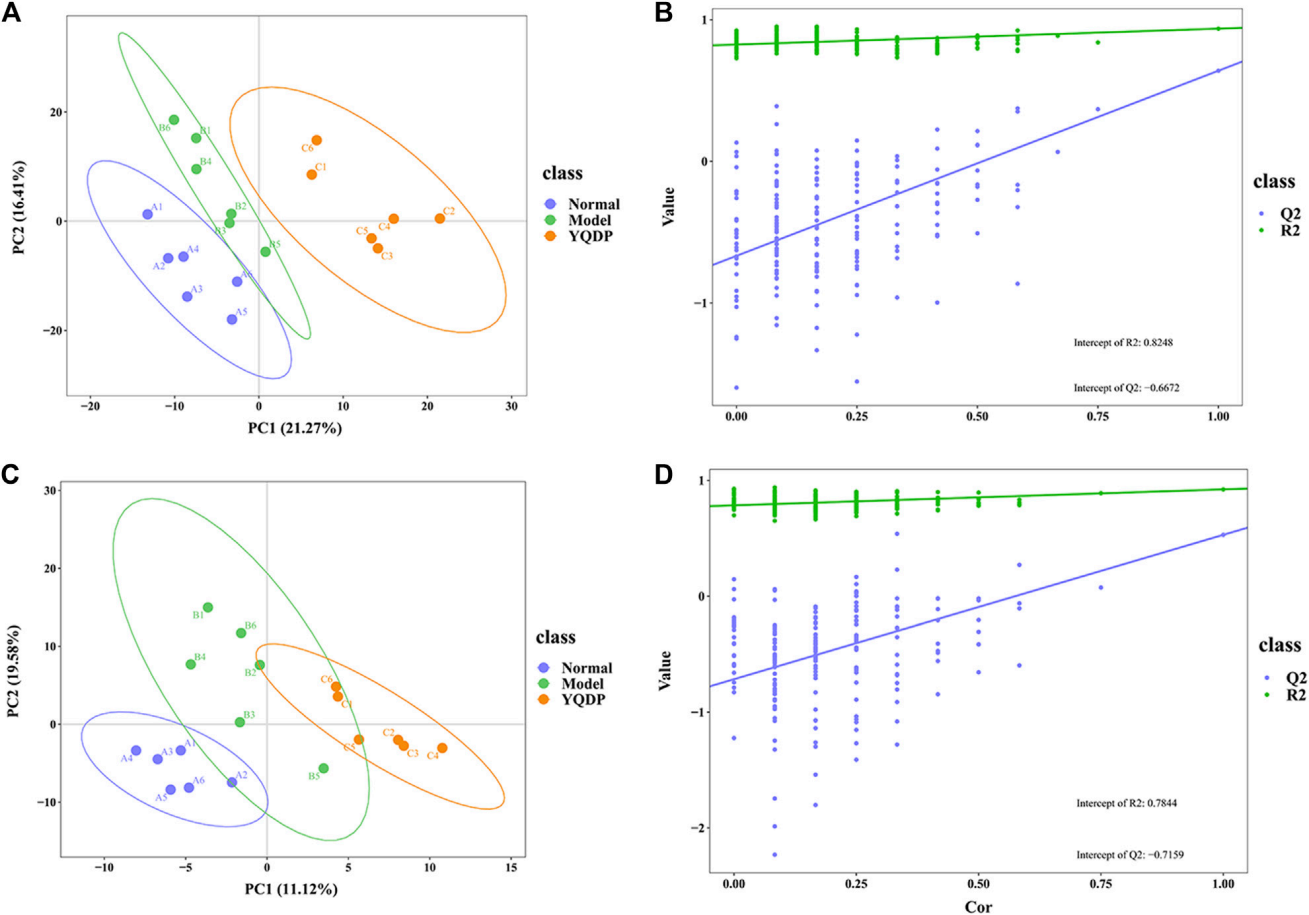
PLS-DA score and model validation of pulmonary metabolites in SMPP mice treated with YQDP. **(A)** Positive PLS-DA. **(B)** Positive PLS-DA Validation. **(C)** Negative PLS-DA. **(D)** Negative PLS-DA Validation.

**FIGURE 7:**
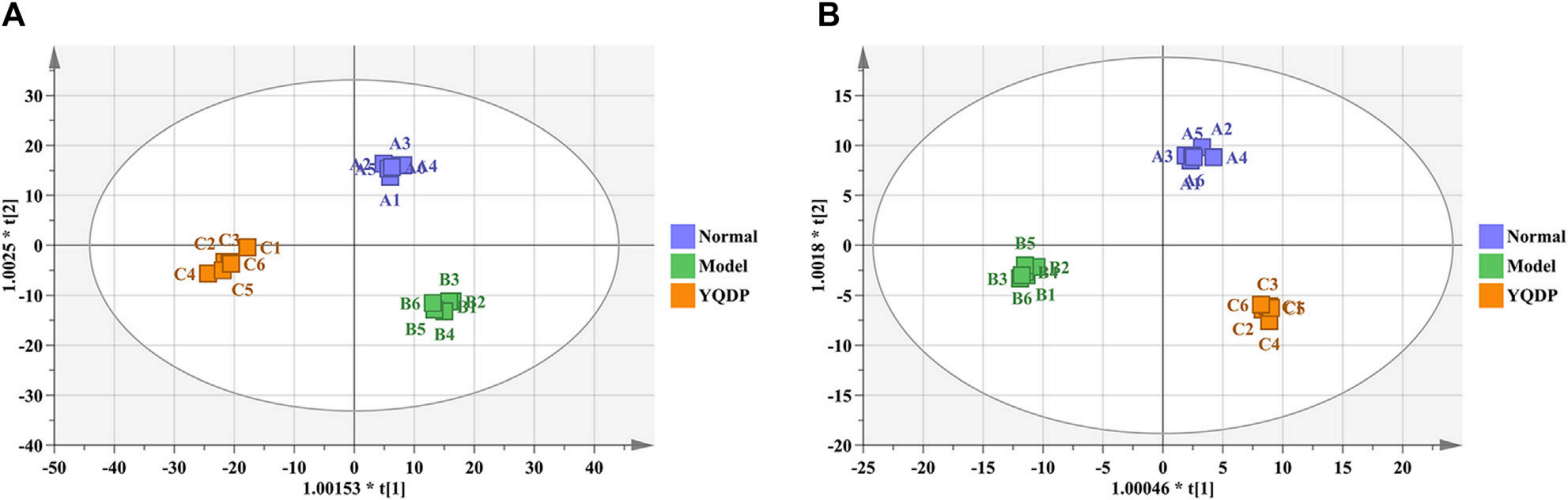
OPLS-DA analysis of pulmonary metabolites in SMPP mice treated with YQDP intervention. **(A)** Positive OPLS-DA. **(B)** Negative OPLS-DA.

**FIGURE 8:**
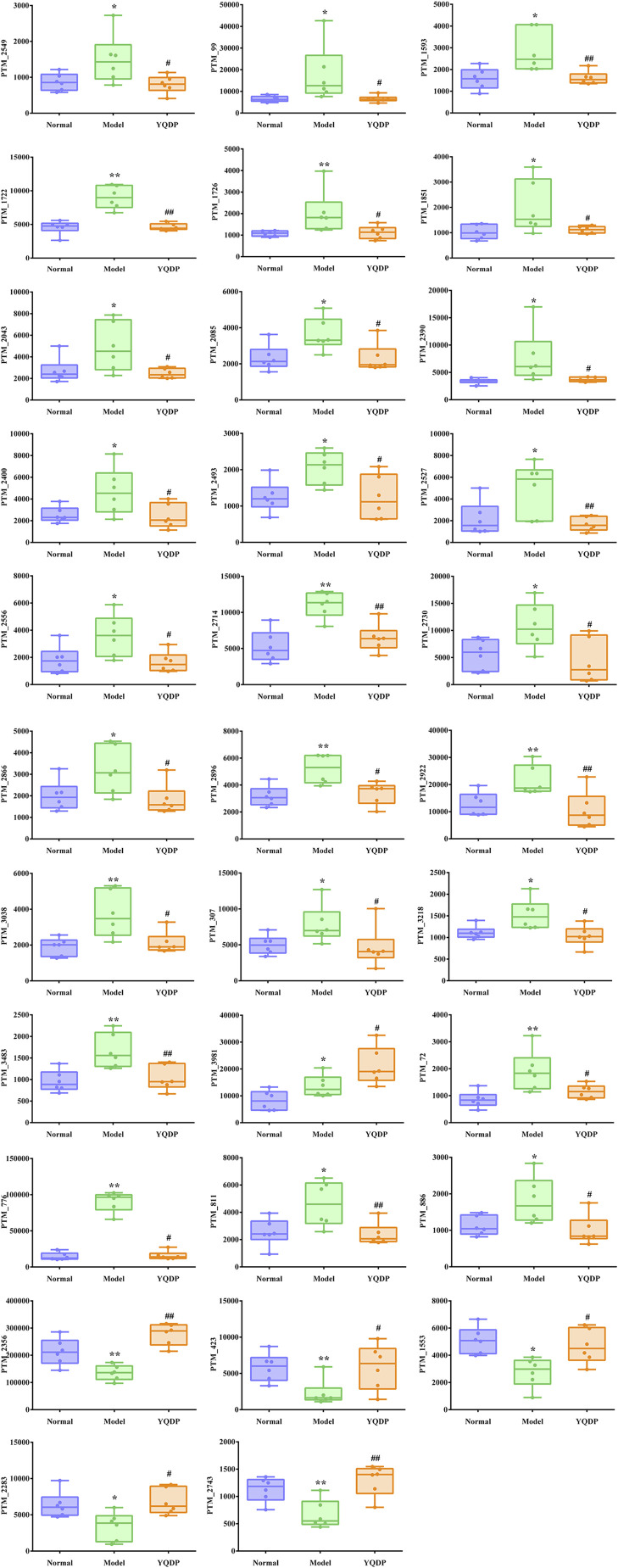
(Continued).

**FIGURE 9:**
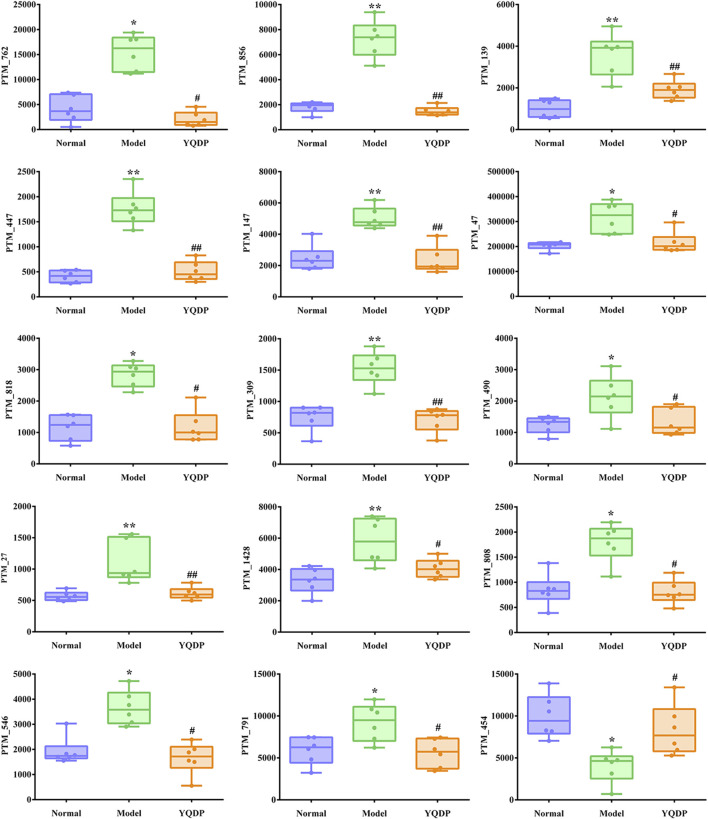
Multiple comparison analysis of differential metabolites in lung tissue under negative ion mode. Compared with normal group, *p < 0.05, **p < 0.01. Compared with model group, #*p* < 0.05, ##*p* < 0.01.

**FIGURE 10:**
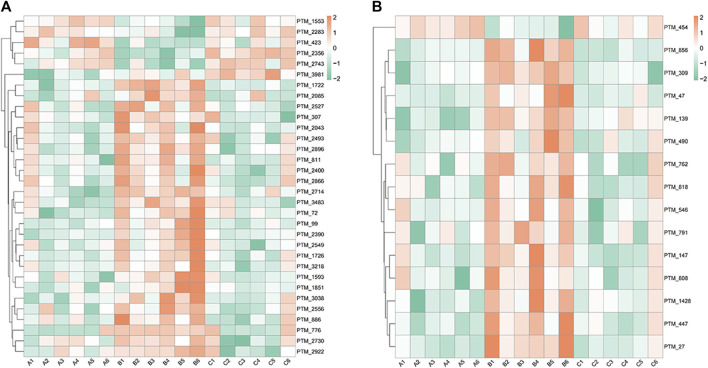
Heat map analysis of differential metabolites in lung tissue. **(A)** Positive. **(B)** Negative.

At 6.208 g/kg, the YQDP group had significant pharmacological effects. In conjunction with the normal group, model group, and YQDP group, metabolomics analysis was carried out. The three groups’ metabolites varied in both positive and negative ion modes, according to PLS-DA analysis. As seen in Figure 6, the P-test findings reveal that there is no overfitting across the group models. Figure 7 further demonstrates a complete separation of the normal, model, and YQDP groups based on OPLS-DA data analysis. In Supplementary Figure S1, the 200 permutation test chart is displayed. Fold Change >1.5 served as the upregulation threshold, Fold Change <1/1.5 served as the significant downregulation threshold, and *t*-test *p* < 0.05 and VIP >1 served as the screening criteria for differential metabolites based on the VIP values of the developed OPLS-DA model. In the positive ion mode, the model group’s lung tissue included 81 metabolites, 64 of which were upregulated and 17 of which were downregulated, significantly different from the normal group’s lung tissue composition. The content of 32 metabolites, including choline phosphate, glutamine, lysine, and L-tyrosinamide, changed following YQDP intervention. In the negative ion mode, the model group’s lung tissue contained significantly less of 49 metabolites (42 upregulated and seven downregulated) than the normal group’s. The levels of 15 metabolites, including Glu Trp, 5-hydroxydecanoate, and 6-thioinosine, varied following YQDP medication. The findings are displayed in Supplementary Tables S2, S3, Figures 8, 9. We displayed the differential metabolite data, as seen in Figure 10, to better comprehend the metabolic variations in the lungs of several groups of mice.

### 3.8 Enrichment analysis of differential metabolite pathways

**FIGURE 11:**
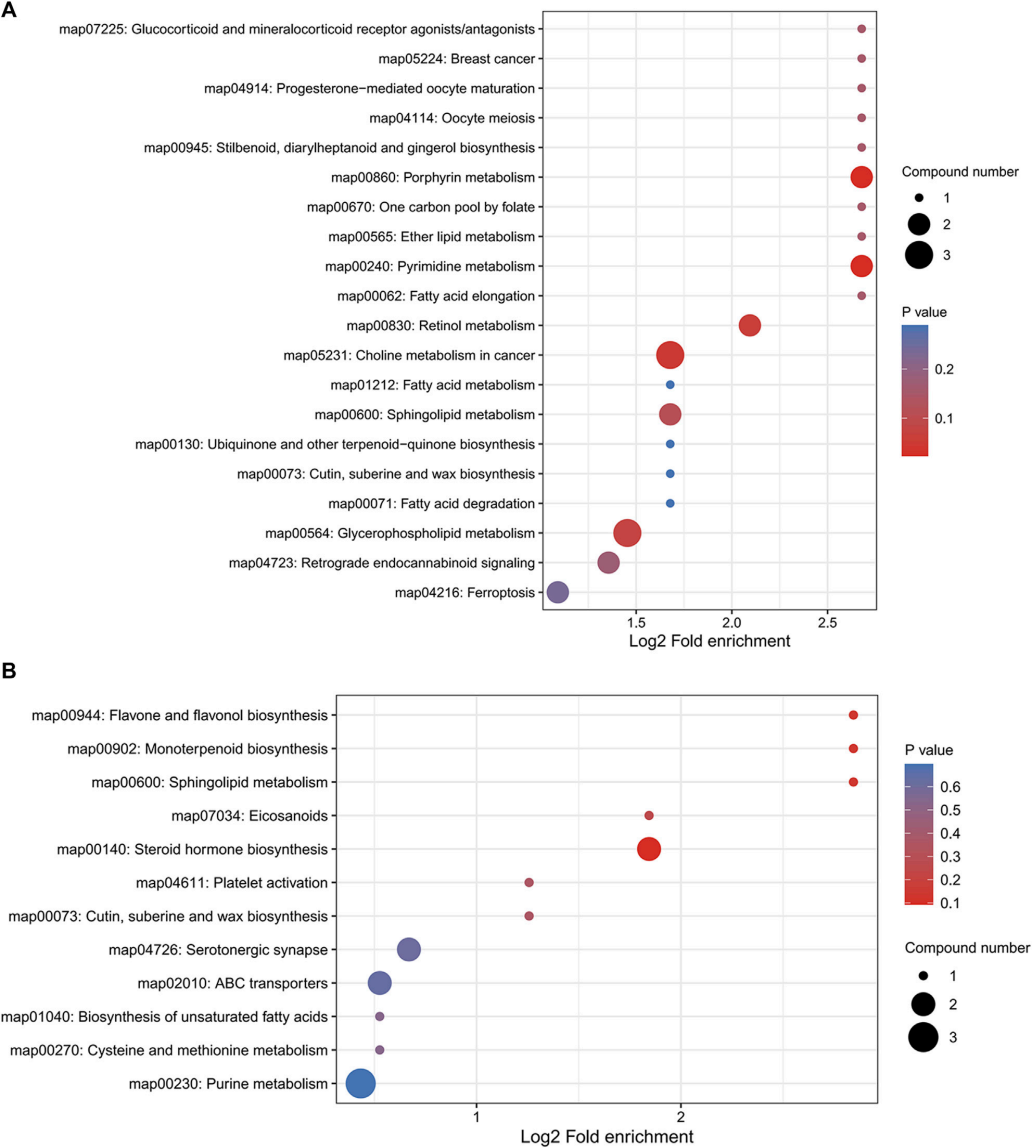
Bubble diagram of KEGG pathway enrichment analysis. **(A)** Positive. **(B)** Negative.

We performed pathway enrichment analysis of metabolites that underwent significant changes in mouse lung tissue after YQDP therapy, as shown in Figure 11. Each circle in the image represents a different metabolic route. As the enrichment factor rises, so does the degree of differential metabolite enrichment in this channel. Based on the results, YQDP may be used to treat SMPP by controlling the metabolism of porphyrins, pyrimidines, cholines, fatty acids, sphingolipids, glycerophospholipids, ferroptosis, steroid hormones, and unsaturated fatty acids.

### 3.9 ELISA for detecting IL-6 and TNF-α expression in serum

**FIGURE 12:**
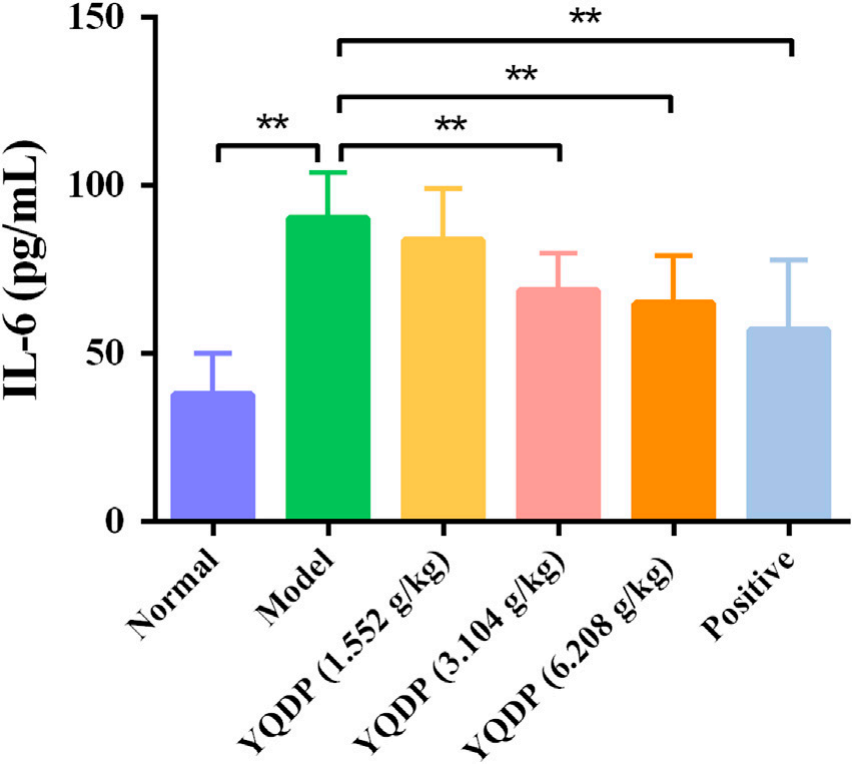
Serum IL-6 expression (pg/mL). **p < 0.01.

**FIGURE 13:**
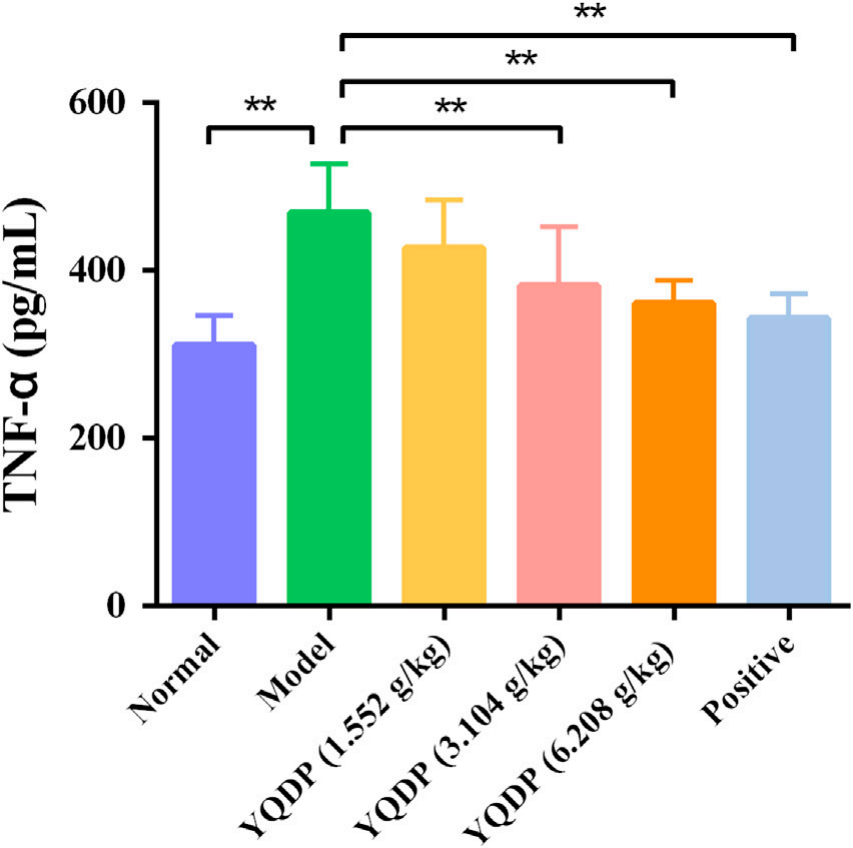
Serum TNF-α expression (pg/mL). **p < 0.01.

The mice in the model group had higher serum expression levels of IL-6 and TNF-α than the mice in the normal group (*p* < 0.01), and the mice in the YQDP administration group had lower expression levels of IL-6 and TNF-α at all concentrations than the mice in the model group (*p* < 0.01). The results are displayed in Figures 12, 13.

### 3.10 Western blot for detecting PI3K, P-PI3K, AKT, P-AKT, NF-κB, P-NF-κB protein expression in lung tissue

**FIGURE 14:**
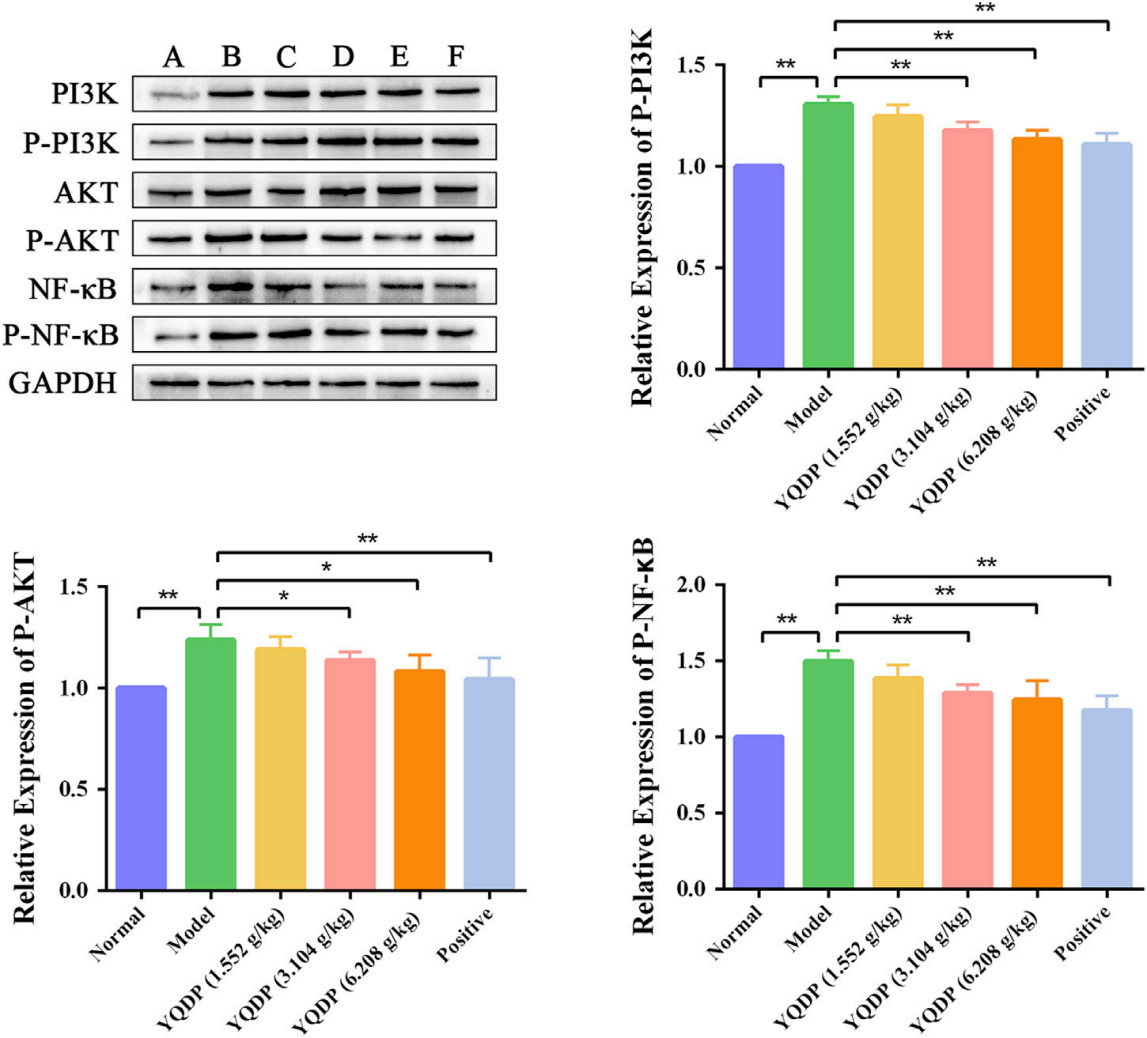
Effect of YQDP on the relative content of PI3K/AKT/NF-κB protein in SMPP mice. **(A)** Normal group. **(B)** Model group. **(C)** YQDP (1.552 g/kg). **(D)** YQDP (3.104 g/kg) group. **(E)** YQDP (6.208 g/kg) group. **(F)** Positive group. *p < 0.05, **p < 0.01.

As demonstrated in Figure 14, the model group mice’s lung tissue showed an increase (*p* < 0.01) in the P-PI3K, P-AKT, and P-NF-κB protein expression ratio when compared to the normal group. In the lung tissue of mice in the positive group and YQDP groups for each dose, the ratio of P-PI3K, P-AKT, and P-NF-κB protein expression reduced (*p* < 0.05) when compared to the model group.

## 4 Discussion

MPP is one of the common community-acquired diseases in children caused by MP infection (Rogozinski et al., 2017), with unremarkable respiratory symptoms and paroxysmal irritating cough as the prominent manifestation of MP infection, accompanied by coughing up and vomiting small amounts of mucus or mucopurulent sputum. In previous studies, our research team applied the theory of “dryness” and the concept of “drying toxins” to treat SMPP using the well-known prescription YQDP from the book “Chonglou Yuyao.” We conducted experiments from multiple angles, including immunoinflammation, epithelial-mesenchymal transition, oxidative stress, and water metabolism, and demonstrated the advantageous targeting of YQDP in intervening in MP infection. First, we conducted a pharmacodynamic component analysis and identified five chemical components in YQDP, including chlorogenic acid, paeoniflorin, forsythoside A, forsythin, and gallic acid. Through the use of experimental techniques such lung tissue pathology, ultrastructure observation, and pulmonary index, we assessed the effectiveness of the model and YQDP. According to the findings, YQDP considerably improved lung tissue pathology and decreased the pulmonary index in SMPP mice. In order to investigate the YQDP’s mechanism of action on SMPP mice further, we used UPLC-MS/MS technology to do a metabolomic study of lung tissue samples from normal, model, and YQDP-treated groups. This study screened 47 differential metabolites that changed after the addition of YQDP, mostly lipids, nucleosides, and fatty acids, which play important roles in inhibiting the immune inflammatory process and participating in multiple inflammatory processes (Das, 2021; Nieto-Garai et al., 2022). The research discovered that while YQDP intervention altered various endogenous metabolites in the model group’s lung tissue, it also altered pathways including the metabolism of α-linolenic acid, sphingolipids, glycerophospholipids, arachidonic acid, and unsaturated fatty acid biosynthesis, which had a therapeutic effect on SMPP mice. α-linolenic acid belongs to the ω-3 polyunsaturated fatty acid family and can generate oxidized lipids under the action of specific enzymes. In terms of inflammation, ω-3 polyunsaturated fatty acids can alleviate and inhibit inflammation (Simopoulos, 2002; Saini and Keum, 2018). The synthesis of phospholipase is an important link that affects the metabolites of glycerophospholipids, and if the content is reduced, it will result in a large number of phospholipid metabolites accumulation. Glycerophospholipid metabolism is achieved through the hydrolysis of phospholipase liaison with the relevant signaling pathways of humoral metabolism, to promote the normal operation of the material and energy metabolism, to maintain metabolic balance and homeostasis (Kouznetsova et al., 2019). Unsaturated fatty acids like arachidonic acid are essential for immunological response, inflammation, and antibacterial activities (Yan et al., 2019; Das, 2021). The study’s findings demonstrated that giving YQDP to SMPP mice decreased the amount of phosphatidylcholine in their lungs, which increased the amount of phosphatidylethanolamine—a material crucial for the metabolism of arachidonic acid in the glycerophospholipid metabolism pathway—and sped up the rate at which phospholipase hydrolyzed the lipid (Zengin et al., 2019). YQDP may be able to treat MP infection-related lipid metabolic abnormalities by controlling glycerophospholipid metabolism. The immune system and inflammatory reactions are greatly influenced by the amounts of endogenous fatty acids and their metabolites. While saturated fatty acids tend to increase inflammation, certain unsaturated fatty acids have anti-inflammatory properties (Rodriguez-Perez et al., 2017; Kotlyarov and Kotlyarova, 2021). Nucleotide production and breakdown are regulated by purine metabolism. The primary cause of multiple organ damage and mortality in SARS-CoV-2-infected severe COVID-19 patients is cytokine storm. Research has revealed a robust association between cytokine storms and dysregulated purine metabolism in COVID-19 patients (Sauer et al., 2012; Zardini et al., 2020), suggesting a direct connection between hyperinflammation and dysregulated purine metabolism in respiratory tract infections. Additionally, studies have demonstrated the regulatory effects of the chemical components in YQDP, including chlorogenic acid, paeoniflorin, gallic acid, glycyrrhizic acid, and forsythoside, on the metabolism of glycerophospholipids, arachidonic acid, and sphingolipids (Zhao, 2018; Cheng et al., 2020; Duan et al., 2020; Feng et al., 2020; Li et al., 2022; Wang et al., 2022).

Phospholipids are essential components of pulmonary surfactants, with the ability to modulate local immune and inflammatory responses, lower alveolar surface tension, preserve alveolar fluid balance, engage in cell membrane protein recognition and conduction, and function as an essential secondary messenger in immune metabolic pathways downstream (Chao et al., 2020). One important modulator of phospholipid conversion is phosphatidylinositide 3-kinases (PI3K), which activates protein kinase B (Akt) signaling pathways that control cellular activity. In order to induce the expression of inflammatory and chemotactic factors, activated Akt can enter the cell nucleus and activate downstream factor NF-κB. This can lead to the infiltration and aggregation of inflammatory cells at the site of inflammation, speeding up the onset and progression of inflammation (Li et al., 2020; Saravia et al., 2020). Following modeling, the model group’s lung tissues had much higher amounts of PI3K/AKT/NF-κB protein than the normal group’s, indicating that the stimulation of inflammatory factors had activated the PI3K/AKT/NF-κB signaling pathway. The ratio of P-PI3K, P-AKT, and P-NF-κB protein expression in lung tissues decreased following YQDP intervention, suggesting that YQDP could prevent the PI3K/AKT/NF-κB signaling pathway from being activated, lessen lung damage, and restore lung function in SMPP animals. The body releases tumor necrosis factor-alpha (TNF-α) as the first cytokine in reaction to a potentially harmful stimulus, and it is essential in starting the inflammatory cascade of cytokines. Co-stimulation of Interleukin-6 (IL-6) production can occur through the combined action of TNF-α and other inflammatory agents. According to certain research, CGA can have anti-inflammatory properties, remove Akt phosphorylation, and decrease the expression of TNF-α and IL-6 (Hou et al., 2016). The experiment’s findings demonstrated that YQDP intervention led to a decrease in the expression of TNF-α and IL-6 in serum, suggesting that YQDP can effectively suppress inflammatory factors’ expression and reduce the inflammatory response.

## 5 Conclusion

In conclusion, YQDP affects SMPP model mice in a certain way. Based on the examination of biological markers and their associated metabolic pathways, conjecture has it that YQDP primarily functions by modulating pathways like the metabolism of α-linolenic acid, sphingolipids, glycerophospholipids, arachidonic acid, and unsaturated fatty acid biosynthesis. Additionally, it may potentially exert a regulatory influence *via* the PI3K/Akt/NF-κB signaling pathway.

## Abbreviation

Akt, Kinase B; AQP5, Aquaporin5; CAP, Community Acquired Pneumonia; CGA, Chlorogenic Acid; ELISA, Enzyme-linked immunosorbent assay; FT, Forsythin; FTA, Forsythoside A; IL-6, Interleukin-6; MP, *Mycoplasma* pneumoniae; MPP, *Mycoplasma* pneumoniae pneumonia; MUC5ac, Mucin 5ac; PAE, Paeonol; PASEF, Parallel accumulation serial fragmentation; PF, Paeoniflorin; PI3K, Phosphatidylinositide 3-kinases; SMPP, Severe *mycoplasma* pneumoniae pneumonia; TCM, Traditional Chinese medicine; TNF-α, Tumor necrosis factor α; YQDP, Yangyin Qingfei Decoction Plus.

## Data Availability

The original contributions presented in the study are included in the article/Supplementary Material, further inquiries can be directed to the corresponding author.

## Abbreviation

Akt, Kinase B; AQP5, Aquaporin5; CAP, Community Acquired Pneumonia; CGA, Chlorogenic Acid; ELISA, Enzyme-linked immunosorbent assay; FT, Forsythin; FTA, Forsythoside A; IL-6, Interleukin-6; MP, *Mycoplasma* pneumoniae; MPP, *Mycoplasma* pneumoniae pneumonia; MUC5ac, Mucin 5ac; PAE, Paeonol; PASEF, Parallel accumulation serial fragmentation; PF, Paeoniflorin; PI3K, Phosphatidylinositide 3-kinases; SMPP, Severe *mycoplasma* pneumoniae pneumonia; TCM, Traditional Chinese medicine; TNF-α, Tumor necrosis factor α; YQDP, Yangyin Qingfei Decoction Plus.
