# Supplementary material for: Research on the metabolic regulation mechanism of Yangyin Qingfei decoction plus in severe pneumonia caused by Mycoplasma pneumoniae in mice
Source: Front Pharmacol. 2024 Apr 17;15:1376812. doi: 10.3389/fphar.2024.1376812 (PMC11061391; doi:10.3389/fphar.2024.1376812)
Supplement: Supplementary file 2 [file Table1.DOCX]

Supplementary Material

**Supplementary Table 1** Results of linear relationship investigation of 5 components in YQDP

| Component | Regression equation | r | Linear range/(μg/mL) |
| --- | --- | --- | --- |
| CGA | *Y* = 1.03×10^5^ X -91.9131 | 0.9997 | 21.366~128.196 |
| PF | *Y* = 4.66×10^4^ X -26.2212 | 0.9996 | 21.717~130.302 |
| FTA | *Y* = 5.45×10^4^ X -49.2581 | 0.9996 | 21.440~128.640 |
| FT | *Y* = 2.25×10^4^ X-7.8746 | 0.9996 | 11.750~70.500 |
| PAE | *Y* = 1.56×10^5^ X -28.6710 | 0.9996 | 6.818~40.907 |

| 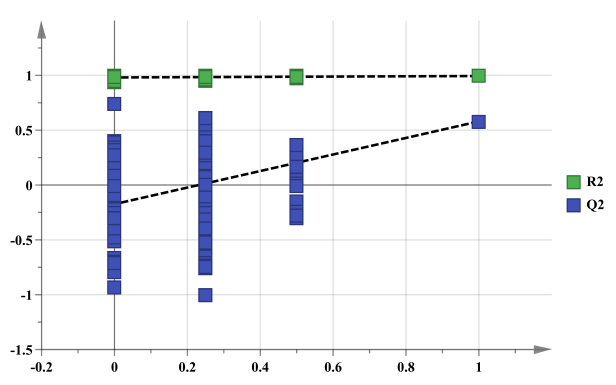 R2(0.0, 0.981), Q2(0.0,-0.174) | 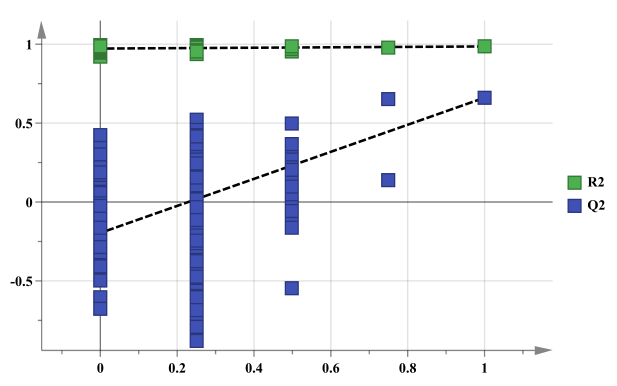 R2(0.0, 0.972), Q2(0.0,-0.196 ) |
| --- | --- |

**Supplementary Figure 1** OPLS-DA 200 permutation test

**Supplementary Table 2** Multiple comparative analysis of differential metabolites in lung tissue under positive ion mode.↑：Up-regulated，*P*＜0.05；↑↑：Up-regulated，*P*＜0.01；↓：Down-regulated，*P*＜0.05；↓↓：Down-regulated，*P*＜0.01.

| No | Differential metabolites | Molecular Formula | Normal vs Model | | | | Model vs YQDP | | | |
| --- | --- | --- | --- | --- | --- | --- | --- | --- | --- | --- |
|  |  |  | *P* value | VIP | FC | Trends | *P* value | VIP | FC | Trends |
| PTM_2549 | Diclobutrazol | C_15_H_19_Cl_2_N_3_O | 0.038 | 1.450 | 1.732 | ↑ | 0.026 | 1.262 | 0.535 | ↓ |
| PTM_99 | URIC ACID | C_5_H_4_N_4_O_3_ | 0.010 | 1.430 | 2.737 | ↑ | 0.011 | 1.124 | 0.369 | ↓ |
| PTM_1593 | SAICAr | C_13_H_18_N_4_O_9_ | 0.010 | 1.747 | 1.814 | ↑ | 0.005 | 1.450 | 0.564 | ↓↓ |
| PTM_1722 | Methyl 2-(propanoylamino)benzoate | C_11_H_13_NO_3_ | 0.000 | 2.242 | 1.977 | ↑↑ | 0.000 | 1.897 | 0.511 | ↓↓ |
| PTM_1726 | Methyl n-acetylanthranilate | C_10_H_11_NO_3_ | 0.010 | 1.534 | 1.891 | ↑↑ | 0.023 | 1.158 | 0.552 | ↓ |
| PTM_1851 | LPC O-22:4 | C_30_H_56_NO_6_P | 0.031 | 1.471 | 1.942 | ↑ | 0.049 | 1.112 | 0.563 | ↓ |
| PTM_2043 | LEU-ILE-ILE | C_24_H_42_N_6_O_5_ | 0.050 | 1.429 | 1.806 | ↑ | 0.027 | 1.348 | 0.500 | ↓ |
| PTM_2085 | Tyrosinamide | C_9_H_12_N_2_O_2_ | 0.014 | 1.697 | 1.559 | ↑ | 0.014 | 1.323 | 0.639 | ↓ |
| PTM_2390 | Glutamyllysine | C_11_H_21_N_3_O_5_ | 0.012 | 1.457 | 2.26 | ↑ | 0.028 | 1.072 | 0.499 | ↓ |
| PTM_2400 | Glu Gln Leu Val Arg | C_27_H_49_N_9_O_9_ | 0.033 | 1.532 | 1.849 | ↑ | 0.035 | 1.272 | 0.513 | ↓ |
| PTM_2493 | Emetine N-oxide | C_29_H_40_N_2_O_5_ | 0.011 | 1.833 | 1.644 | ↑ | 0.035 | 1.406 | 0.601 | ↓ |
| PTM_2527 | Docosan-1-amine | C_22_H_47_N | 0.038 | 1.544 | 2.282 | ↑ | 0.008 | 1.539 | 0.342 | ↓↓ |
| PTM_2556 | 2-(2-Aminoethyl)pyridine | C_7_H_10_N_2_ | 0.031 | 1.549 | 1.972 | ↑ | 0.012 | 1.422 | 0.456 | ↓ |
| PTM_2714 | Austroinulin | C_20_H_34_O_3_ | 0.001 | 2.187 | 2.104 | ↑↑ | 0.002 | 1.722 | 0.584 | ↓↓ |
| PTM_2730 | Asn-Arg-Arg | C_16_H_32_N_10_O_5_ | 0.036 | 1.617 | 1.933 | ↑ | 0.033 | 1.415 | 0.400 | ↓ |
| PTM_2866 | 9H-12(13)-EpODE | C_18_H_30_O_4_ | 0.045 | 1.480 | 1.586 | ↑ | 0.018 | 1.349 | 0.570 | ↓ |
| PTM_2896 | Atearic acid | C_18_H_36_O_2_ | 0.003 | 1.975 | 1.641 | ↑↑ | 0.013 | 1.513 | 0.657 | ↓ |
| PTM_2922 | (8Z,11Z)-14,15-Epoxy-8,11-icosadienoic acid | C_21_H_36_O_3_ | 0.008 | 1.802 | 1.691 | ↑↑ | 0.010 | 1.507 | 0.491 | ↓↓ |
| PTM_3038 | 5-methoxy-2-methyl-6-[(E)-2-nitroethenyl]-2,3-dihydro-1-benzofuran | C_12_H_13_NO_4_ | 0.006 | 1.835 | 1.946 | ↑↑ | 0.013 | 1.384 | 0.571 | ↓ |
| PTM_307 | SM 8:1 | C_35_H_59_N_2_O_6_P | 0.024 | 1.549 | 1.569 | ↑ | 0.041 | 1.224 | 0.595 | ↓ |
| PTM_3218 | 3-cyclopropyl-5-(2-pyrrolidin-3-yloxyphenyl)-1,2,4-oxadiazole | C_15_H_17_N_3_O_2_ | 0.047 | 1.266 | 1.583 | ↑ | 0.030 | 1.089 | 0.585 | ↓ |
| PTM_3483 | 13-Oxo-8(14)-podocarpen-18-oic acid | C_17_H_24_O_3_ | 0.003 | 1.962 | 1.737 | ↑↑ | 0.009 | 1.514 | 0.622 | ↓↓ |
| PTM_3981 | (2E)-2-[2-(1,2,4a,5-Tetramethyl-1,2,3,4,4a,7,8,8a-octahydro-1-naphthalenyl)ethyl]-2-butenedioic acid | C_20_H_30_O_4_ | 0.031 | 1.566 | 1.645 | ↑ | 0.032 | 1.264 | 1.549 | ↑ |
| PTM_72 | Val Cys Ser Arg | C_17_H_33_N_7_O_6_S | 0.004 | 1.840 | 2.225 | ↑↑ | 0.025 | 1.245 | 0.605 | ↓ |
| PTM_776 | PG(16:0/0:0) | C_22_H_45_O_9_P | 0.001 | 2.100 | 3.584 | ↑↑ | 0.021 | 1.305 | 0.487 | ↓ |
| PTM_811 | PE O-18:2_18:1 | C_41_H_78_NO_7_P | 0.033 | 1.654 | 1.819 | ↑ | 0.008 | 1.464 | 0.515 | ↓↓ |
| PTM_886 | PE(18:2(9Z,12Z)/PGE2) | C_43_H_74_NO_11_P | 0.021 | 1.592 | 1.621 | ↑ | 0.013 | 1.369 | 0.550 | ↓ |
| PTM_2356 | Choline Alfoscerate | C_8_H_20_NO_6_P | 0.006 | 1.875 | 0.639 | ↓↓ | 0.000 | 1.827 | 2.042 | ↑↑ |
| PTM_423 | Progesterone | C_21_H_30_O_2_ | 0.001 | 1.826 | 0.159 | ↓↓ | 0.031 | 1.437 | 2.572 | ↑ |
| PTM_1553 | 7-Chloro-4-phenyl-2,3,4,5-tetrahydro-1H-1,5-benzodiazepine-2-carbonitrile | C_16_H_14_ClN_3_ | 0.013 | 2.016 | 0.539 | ↓ | 0.037 | 1.419 | 1.701 | ↑ |
| PTM_2283 | His Ile Thr | C_16_H_27_N_5_O_5_ | 0.034 | 1.692 | 0.538 | ↓ | 0.024 | 1.515 | 1.981 | ↑ |
| PTM_2743 | Arg Pro Asp Val | C_20_H_35_N_7_O_7_ | 0.008 | 1.867 | 0.591 | ↓↓ | 0.003 | 1.695 | 1.946 | ↑↑ |

**Supplementary Table 3** Multiple comparative analysis of differential metabolites in lung tissue under negative ion mode. ↑：Up-regulated，*P*＜0.05；↑↑：Up-regulated，*P*＜0.01；↓：Down-regulated，*P*＜0.05；↓↓：Down-regulated，*P*＜0.01.

| No | Differential metabolites | Molecular Formula | Normal vs Model | | | | Model vs YQDP | | | |
| --- | --- | --- | --- | --- | --- | --- | --- | --- | --- | --- |
|  |  |  | P value | VIP | FC | Trends | P value | VIP | FC | Trends |
| PTM_762 | 6-Thioinosine | C_10_H_12_N_4_O_4_S | 5.280 | 0.020 | 1.419 | ↑ | 0.192 | 0.014 | 1.430 | ↓ |
| PTM_856 | 3-O-Feruloylquinic Acid | C_17_H_20_O_9_ | 4.561 | 0.001 | 1.435 | ↑↑ | 0.180 | 0.000 | 1.530 | ↓↓ |
| PTM_139 | Secologanoside | C_16_H_22_O_11_ | 3.607 | 0.000 | 1.883 | ↑↑ | 0.529 | 0.003 | 1.710 | ↓↓ |
| PTM_447 | Hesperetin Dihydrochalcone | C_16_H_16_O_6_ | 3.073 | 0.003 | 1.691 | ↑↑ | 0.403 | 0.012 | 1.562 | ↓ |
| PTM_147 | Rutin | C_27_H_30_O_16_ | 2.233 | 0.005 | 1.259 | ↑↑ | 0.420 | 0.004 | 1.365 | ↓↓ |
| PTM_47 | Uric Acid | C_5_H_4_N_4_O_3_ | 2.171 | 0.022 | 1.547 | ↑ | 0.496 | 0.019 | 1.424 | ↓ |
| PTM_818 | 5'-Fluoro-2'-hydroxy-4-methylchalcone | C_16_H_13_FO_2_ | 2.160 | 0.014 | 1.623 | ↑ | 0.468 | 0.013 | 1.651 | ↓ |
| PTM_309 | TAPSO | C_7_H_17_NO_7_S | 2.035 | 0.001 | 1.861 | ↑↑ | 0.466 | 0.000 | 1.960 | ↓↓ |
| PTM_490 | Oglufanide | C_16_H_19_N_3_O_5_ | 1.981 | 0.024 | 1.352 | ↑ | 0.536 | 0.041 | 1.284 | ↓ |
| PTM_27 | Volemitol | C_7_H_16_O_7_ | 1.942 | 0.001 | 1.824 | ↑↑ | 0.557 | 0.002 | 1.730 | ↓↓ |
| PTM_1428 | (5E)-5-(3,4-Dimethoxybenzylidene)-4-hydroxy-1,3-thiazol-2(5H)-one | C_12_H_11_NO_4_S | 1.922 | 0.006 | 1.540 | ↑↑ | 0.642 | 0.037 | 1.341 | ↓ |
| PTM_808 | 5-(4-Chloroanilino)-5-oxopentanoic acid | C_11_H_12_ClNO_3_ | 1.666 | 0.042 | 1.259 | ↑ | 0.569 | 0.018 | 1.360 | ↓ |
| PTM_546 | Estrone glucuronide | C_24_H_30_O_8_ | 1.650 | 0.027 | 1.477 | ↑ | 0.519 | 0.025 | 1.607 | ↓ |
| PTM_791 | 5-Hydroxydecanoate | C_10_H_20_O_3_ | 1.558 | 0.021 | 1.551 | ↑ | 0.605 | 0.011 | 1.494 | ↓ |
| PTM_454 | Guanosine | C_10_H_13_N_5_O_5_ | 0.406 | 0.011 | 1.863 | ↓ | 2.063 | 0.037 | 1.537 | ↑ |
